# Divergent effects of canonical and non‐canonical TGF‐β signalling on mixed contractile‐synthetic smooth muscle cell phenotype in human Marfan syndrome aortic root aneurysms

**DOI:** 10.1111/jcmm.14921

**Published:** 2019-12-30

**Authors:** Albert J. Pedroza, Tiffany Koyano, Jeffrey Trojan, Adam Rubin, Itai Palmon, Kevin Jaatinen, Grayson Burdon, Paul Chang, Yasushi Tashima, Jason Z. Cui, Gerry Berry, Cristiana Iosef, Michael P. Fischbein

**Affiliations:** ^1^ Department of Cardiothoracic Surgery Stanford University School of Medicine Stanford California; ^2^ Stanford University School of Medicine Stanford California; ^3^ Department of Pathology Stanford University School of Medicine Stanford California

**Keywords:** aortic aneurysm, Marfan syndrome, smooth muscle cell phenotype

## Abstract

Aortic root aneurysm formation is a cardinal feature of Marfan syndrome (MFS) and likely TGF‐β driven via Smad (canonical) and ERK (non‐canonical) signalling. The current study assesses human MFS vascular smooth muscle cell (SMC) phenotype, focusing on individual contributions by Smad and ERK, with Notch3 signalling identified as a novel compensatory mechanism against TGF‐β‐driven pathology. Although significant ERK activation and mixed contractile gene expression patterns were observed by traditional analysis, this did not directly correlate with the anatomic site of the aneurysm. Smooth muscle cell phenotypic changes were TGF‐β‐dependent and opposed by ERK in vitro*,* implicating the canonical Smad pathway. Bulk SMC RNA sequencing after ERK inhibition showed that ERK modulates cell proliferation, apoptosis, inflammation, and Notch signalling *via* Notch3 in MFS*.* Reversing Notch3 overexpression with siRNA demonstrated that Notch3 promotes several protective remodelling pathways, including increased SMC proliferation, decreased apoptosis and reduced matrix metalloproteinase activity, in vitro. In conclusion, in human MFS aortic SMCs: (a) ERK activation is enhanced but not specific to the site of aneurysm formation; (b) ERK opposes TGF‐β‐dependent negative effects on SMC phenotype; (c) multiple distinct SMC subtypes contribute to a ‘mixed’ contractile‐synthetic phenotype in MFS aortic aneurysm; and (d) ERK drives Notch3 overexpression, a potential pathway for tissue remodelling in response to aneurysm formation.

## 1 INTRODUCTION

Marfan syndrome (MFS) is an inheritable connective tissue disorder resulting from fibrillin‐1 (Fbn1) mutations with an incidence of 1 in 5000 individuals.^1^, ^2^ Aortic root aneurysm formation and ensuing dissection accounts for early mortality if not surgically repaired prophylactically.^3^ Transforming growth factor‐beta (TGF‐β) signalling is central to MFS aortic aneurysm pathogenesis.^4^ Despite enhanced TGF‐β signalling systemically, MFS patients classically develop focal aortic root aneurysms with preserved dimension of the adjacent ascending aorta.^5^ We generally hypothesize that distinct embryonic origins of thoracic aortic segments^6^ (second heart field, neural crest, or paraxial mesoderm) may govern variable aortic smooth muscle cell (SMC) response to enhanced TGF‐β and explain this predisposition to aortic root dilatation, although hemodynamic and other biomechanical factors have been proposed as mediators of region‐specific changes as well.^7^

Paradoxically, TGF‐β signalling seems to have a variable role depending on aortic developmental stage: critical for early normal aortic wall development,^8^ while pathologic when enhanced during late aneurysm formation.^9^ Multiple studies^10^, ^11^, ^12^ of compound mutant murine models combining Fbn1 mutations with knockout of TGF‐β signalling proteins have demonstrated that this dynamic role of TGF‐β seems to depend on developmental age, magnitude, receptor expression, aortic embryologic origin and disease state. TGF‐β signalling proceeds through both canonical (Smad‐dependent) and non‐canonical, extracellular signal‐regulated kinase (ERK), c‐Jun N‐terminal kinase (JNK) and p38 mitogen‐activated protein kinase (p38 MAPK)‐dependent pathways.^13^, ^14^ Holm et al^15^ illustrated that selective ERK1/2 blockade reduces aneurysm development in a murine MFS model, whereas compound MFS Fbn1^C1039G/+^ mutants with Smad4 knockout demonstrated worsened aneurysm severity, suggesting that these divergent TGF‐β pathways may have opposing effects. It remains unclear how activation of these pathways results in aortic aneurysmal disease. Moreover, the fidelity of this hypothesized mechanism in human disease has not been firmly established. Understanding the pathophysiology of the downstream TGF‐β signalling pathways would provide a theoretical framework for the development of targeted therapeutics aimed at slowing or preventing aneurysm growth.

Smooth muscle cell dysfunction (modulation from contractile to the dedifferentiated synthetic phenotype) is known to contribute to aortic aneurysm development in a variety of hereditary aneurysm phenotypes.^16^, ^17^, ^18^ Crosas‐Molist et al^19^ demonstrated that SMCs in MFS patients actually display *increased* expression of contractile proteins concurrent with enhanced collagen expression, both of which could be reversed with TGF‐β blockade in vitro. Following TGF‐β activation, both Smad and ERK modulate gene expression and SMC phenotype in arterial development and disease,^20^ but their individual effects on SMCs in MFS remain undefined. In this study, we systematically dissect the effects of ERK signalling downstream of TGF‐β to: (a) compare ERK signalling in aneurysmal aortic root vs non‐dilated ascending aortic specimens from human MFS patients; (b) analyse the relative contributions of Smad and ERK to known SMC phenotype changes in MFS; and (c) identify downstream ERK‐dependent pathways in primary cultured MFS SMCs to further elucidate the role of TGF‐β signalling during aneurysm formation. Intriguingly, we report that ERK drives Notch3 overexpression, a potential protective pathway for tissue remodelling in response to MFS aneurysm formation. Four Notch receptors (Notch 1‐4) have been described in humans and represent large transmembrane proteins that bind ligands expressed on adjacent cells.^21^ Because Notch plays a key role in neural crest migration and SMC differentiation during ascending aorta/aortic arch development, abnormal signalling may predispose to aneurysm formation.^22^ Although Notch signalling has not been studied in MFS, Notch1 gene mutations have been reported in patients with bicuspid aortic valves (BAV) and BAV aortopathy. Notch 1‐4 mRNA levels were significantly decreased in BAV aortic specimens compared to normal tricuspid aortic valve aortas.^23^ Similarly, reduced Notch 1 and 3 gene expression levels were reported in human abdominal aortic aneurysm samples.^24^ We hypothesize the Notch pathway incites productive tissue remodelling in response to MFS aneurysm formation and affords a provocative avenue for therapeutic intervention via forced Notch3 overexpression.

## 2 MATERIALS AND METHODS

### 2.1 Human studies

The Stanford Institutional Review Board (IRB) approved experiments involving human specimens. All patients included in this study gave informed consent for tissue banking and participation in human subject studies during elective cardiac surgery cases. Blanket research consent was obtained from surrogate decision‐makers for all included organ donor controls by the referring organ procurement organization.

### 2.2 Tissue handling

Fresh surgical specimens were collected within 30 minutes of excision, dissected to remove adventitial tissue, and snap‐frozen in liquid nitrogen. For molecular assays, tissue samples were thawed and remaining adventitial and intimal layers removed. The tissue was snap‐frozen prior to lysis for downstream analysis.

### 2.3 Protein isolation and processing

Isolated medial aortic tissue was suspended in RIPA lysis buffer (MilliporeSigma, St. Louis, MO) supplemented with pan‐protease and phosphatase inhibitor cocktail (MilliporeSigma) and disrupted with a rotor/stator homogenizer, snap‐frozen and homogenized again. Cultured SMCs monolayers were treated with Trypsin (TrypleE, Gibco), cells were pelleted in a microcentrifuge, washed in PBS and lysed with RIPA buffer. Lysates were allowed to dissociate on a rotator at 4**°**C for 60 minutes, then centrifuged to pellet insoluble tissue debris. The supernatant was collected and subjected to protein content quantification by BCA assay kit (ThermoFisher Scientific).

### 2.4 Wes semi‐quantitative protein immunoblotting

Protein lysates from tissue and in vitro cell culture lines were processed for use on *Wes* Simple Western assays according to manufacturer protocols (Protein Simple). Samples were mixed with Simple Western Sample Master Mix (80 mmol/L DTT, 2× sample buffer, 2× fluorescence standard) and denatured. The Simple Western kit plate was loaded with denatured samples, primary antibody, HRP‐conjugated anti‐rabbit antibody, luminol‐peroxide substrate and wash buffers. The proprietary capillary‐based separation system was utilized to automatically load, separate, immobilize and immunoprobe protein lysates for proteins of interest using HRP‐mediated chemiluminescence. The chemiluminescent signal was detected using the system's built‐in CCD camera and analysed for signal intensity using accompanying Compass software. Band intensity was used to generate a traditional Western blot lane. Primary antibodies were titrated to determine optimal protein and primary antibody concentration. Antibodies for pERK1/2 (1:100 dilution, rabbit monoclonal, #4695), pSMAD2/3 (1:50 dilution, rabbit monoclonal, #8828), vinculin (1:150 dilution, rabbit polyclonal #4650) and Notch3 (1:100 dilution, rabbit monoclonal #5276) were purchased from Cell Signaling Technology.

### 2.5 RNA isolation and processing

Total RNA was extracted from frozen tissue using TRIzol (ThermoFisher Scientific). Cultured SMCs were trypsinized, pelleted in a microcentrifuge, washed in PBS and processed for RNA using the GeneJET RNA Purification Kit (ThermoFisher, Scientific). RNA was purified using the GeneJET RNA Cleanup and Concentration micro kit (ThermoFisher Scientific). RNA concentration was calculated using a NanoDrop spectrophotometer and quality determined by analysing the A_260/280_ ratio for values > 1.9.

### 2.6 cDNA synthesis and quantitative RT‐PCR

RNA samples were normalized by concentration and subjected to cDNA synthesis using the Maxima First Strand cDNA Synthesis Kit (ThermoFisher Scientific). 100‐400 ng of RNA was used for cDNA synthesis depending on application and then diluted for RT‐PCR reactions. All probes for RT‐PCR were purchased from Life Technologies TaqMan gene expression assays. Gene expression experiments were performed in 384‐well plates and analysed on the ViiA‐7 system (ThermoFisher Scientific). Gene expression values were calculated using the ΔCT method utilizing 18S as an endogenous control. Commercially available, verified RT‐PCR primers were purchased for all genes (ThermoFisher Scientific).

### 2.7 Vascular SMC isolation and culture

Aortic tissue samples were placed into Waymouth's Medium for Aortic Biopsy supplemented with 3% Sodium Bicarbonate, 1.25% 200 mmol/L l‐Glutamine (100×), 1% Antibiotic‐Antimycotic (Anti‐Anti) (100×), 1% 1 mol/L 4‐(2‐hydroxyethyl)‐1‐piperazineethanesulfonic acid (HEPES) (1×) and 1% Minimum Essential Medium Non‐Essential Amino Acid Solution (MEM NEAA) (100×) (ThermoFisher Scientific) and transferred to a cell culture hood. Intima and adventitia layers were discarded, while the tunica media layer was placed into a culture dish containing 5 mL of aortic biopsy media with 0.1 mg/mL Collagenase Type I and 0.025 mg/mL Soybean Trypsin Inhibitor. The aortic tissue was minced and the dish placed in an incubator overnight at 37**°**C and 5% CO_2_. Foetal bovine serum (Lonza) was added after incubation to arrest digestion. The contents were collected and centrifuged; the supernatant was removed and the pellet was released with 4 mL of SMC culture media that consisted of Smooth Muscle Basal Media (Lonza), 10% FBS, SmGm‐2 SingleQuot kit (Insulin, rhFGF, rhEGF) (#CC3182 Lonza), 1% Anti‐Anti 100×, 1% 200 mmol/L l‐Glutamine (100×), 1% 100 mmol/L Sodium Pyruvate (100×), and 2% 1 mol/L HEPES (1×). Cell lines were maintained in SMC media and passaged once culture flasks reached 70%‐80% confluency.

### 2.8 SMC culture in vitro assays

Human aortic SMC lines were used for in vitro assays between passages 4‐6. Smooth muscle cells were grown to 70% confluency in SMC media and split into 6‐well plates. Once appropriate cell density was reached, SMCs were starved of serum and growth factors overnight and subjected to treatment with TGF‐β1 (10 ng/mL; #100‐B; R&D Systems) and either (a) ERK activation inhibitor PD98059 (20 μm; #9900; Cell Signaling Technology, Danvers, MA), (b) activin receptor‐like Kinase (ALK) receptor inhibitor (Smad inhibitor) SB‐431542 (10 μmol/L; #S4317; MilliporeSigma, St. Louis, MO) or (c) control carrier for 24 hours. For ERK inhibition prior to RNA sequencing, cells were starved overnight and treated with PD98059 at 20 μmol/L for 24 hours.

### 2.9 Single‐cell RNA sequencing

Human aortic root SMC lines were serum starved overnight then treated with differentiation media (1% FBS). The cells were then treated with Trypsin (TrypleE, Gibco) and resuspended into a single‐cell suspension in PBS with 0.4% BSA. Single‐cell capture and cDNA library synthesis were completed at the Stanford Functional Genomics Facility according to manufacturer specifications using a 10× Genomics microfluidic chip (10× Genomics, Pleasanton, CA). Libraries were sequenced on an Illumina HiSeq 4000 instrument.

### 2.10 mRNA sequencing and analysis

Total RNA pools from SMC lines treated with either (1) PD98059 or (2) vehicle control were prepared for mRNA sequencing using the TruSeq Stranded mRNA Library Prep Kit (Illumina; RSS‐122‐2103; San Diego, CA). Samples were sequenced by the Stanford Genome Sequence Servicing Center (Stanford, CA) on the NextSeq 550 high output platform (Illumina) with single‐end reads of 150 bp. All samples were checked for quality using a Bioanalyzer prior to library preparation. Single‐end reads were aligned to Hg19 genome using TopHat2 software (http://ccb.jhu.edu/software/tophat/index.shtml) with default settings. HTSeq was then used to determine read counts supporting expression of genes from RefSeq Hg19 (the htseq‐count function). These values were log‐transformed and normalized as counts per million reads as input for QuSage (http://bioconductor.org/packages/release/bioc/html/qusage.html). The two‐way paired group response for Donor control or MFS patient samples was used to determine gene sets with patient‐specific responses to PD98059 treatment. Single‐gene‐level data for ERK inhibitor responses were generated by cross‐referencing the differentially expressed genes for each sample group to create an MFS aortic root‐specific gene set consisting of 228 genes. The DAVID bioinformatics database was used to cluster these genes by biologic function and screened for pathways of interest.

### 2.11 Notch3 siRNA knockdown

Adherent SMCs of 60% confluence in 6 well plates were washed with PBS and transfected with (a) Notch3 siRNA (10 μmol/L) or (b) FAM‐labelled scrambled control siRNA and Lipofectamine (ThermoFisher Scientific) for 4 hours in serum‐free hSMC media. Washed cells were imaged using fluorescence to confirm transfection success. Cells were cultured in hSMC media for 48 hours prior to further assays.

### 2.12 MTT proliferation assay

Smooth muscle cells were seeded in a 96‐well optical plate after siRNA transfection and given (a) standard or (b) serum‐free hSMC media for 48 hours. The MTT assay (Promega) was performed according to the manufacturer's protocol and the absorbance read at 570 nm.

### 2.13 Annexin V Apoptosis Assay (Fluorescence‐Activated Cell Sorting)

Transfected SMCs were trypsinized and counted after 48 hours. Smooth muscle cells (10 000) were incubated with fluorescein isothiocyanate (FITC), propidium iodide (PI) and Annexin V Binding Buffer according to the FITC Annexin V/Dead Cell Apoptosis Detection Kit (Life Technologies Corporation). Staurosporine (47 ng/mL, Enzo Life Science) was added 24 hours prior to analysis as a positive control and (a) unstained, (b) FITC and (c PI were used as negative controls. Cells were analysed with flow cytometry on a DxP FACScan (BD Biosciences, Haryana, India) using blue and yellow lasers. Data were analysed with FlowJo (Ashland, Oregon) version 10.

### 2.14 Total elastin quantification assay

Total cell lysates after siRNA transection and 48 hours incubation were isolated from 200 000 cells using RIPA Buffer (MilliporeSigma). Total bound elastin was quantified using the Fastin Elastin Kit (Biocolor; Carrickfergus, UK) using manufacturer protocols. A standard curve was generated with provided purified elastin for quantification of total sample elastin.

### 2.15 Gelatin zymography

Conditioned media from siRNA‐transfected SMCs incubated in hSMC media for 48 hours was collected and concentrated using Amicon Ultra‐4 centrifugal filter tubes (Ultracel‐30K) (MilliporeSigma). The pro and active forms of MMP‐2 were evaluated with commercially available Gelatin Zymography Kit (Cosmobio) using 10 μL of concentrated supernatant according to manufacturer protocols. Band density was calculated using ImageJ.

### 2.16 Statistical analysis

Statistics were calculated using R (https://www.r-project.org/). Assays were analysed using two‐way paired Student's *t* test when comparing anatomic segments within patients and treated samples to control for in vitro experiments and non‐paired Student's *t* test for comparisons of tissue from different individuals. Bonferroni correction was used where applicable to adjust for multiple comparisons. *P* values <.05 were considered significant.

## 3 RESULTS

### 3.1 ERK signalling is enhanced in both aneurysmal and normal calibre aortic specimens from MFS patients

**Figure 1:**
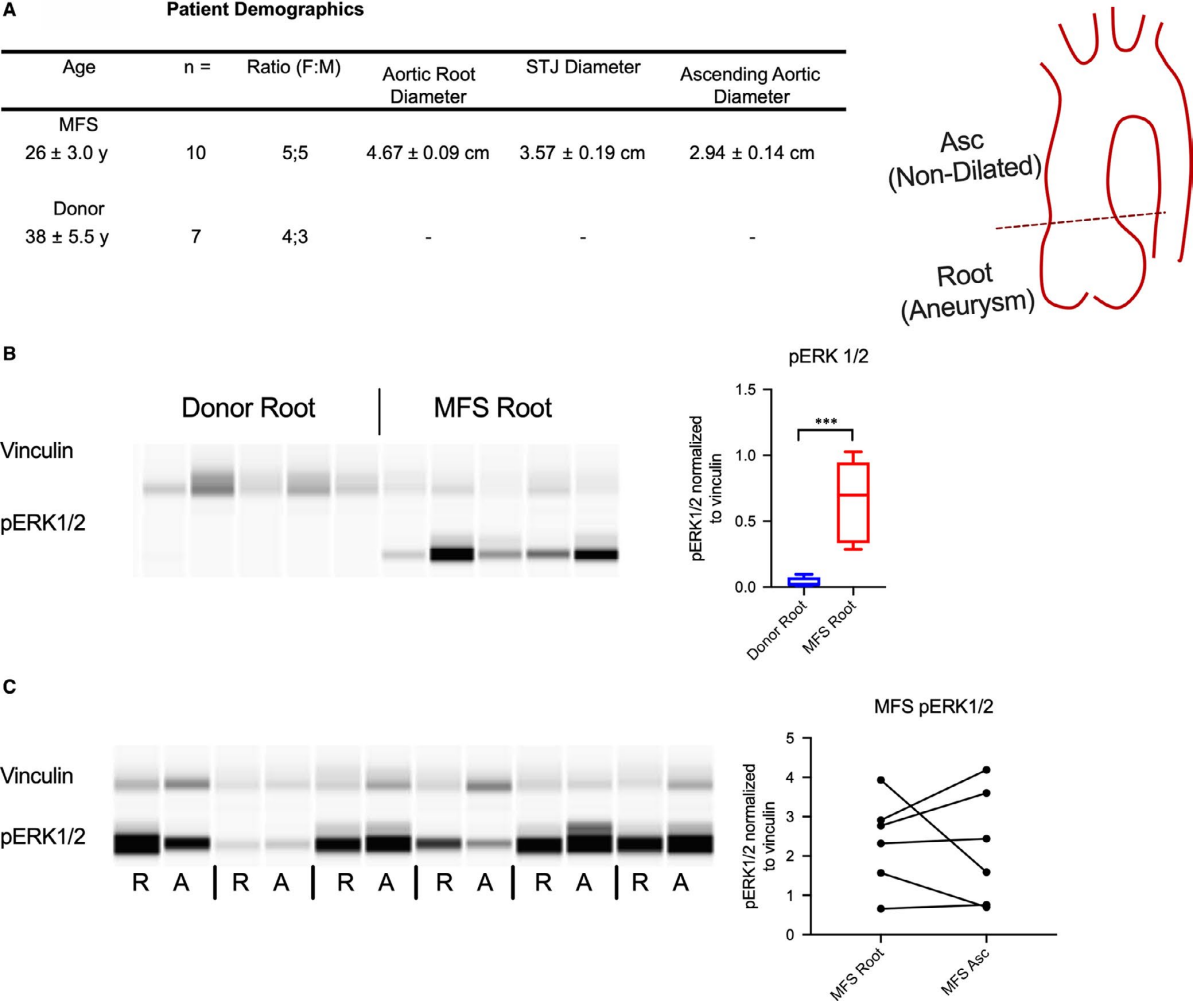
ERK signalling is enhanced in MFS aortic samples. A, Demographics and aortic diameters for included patients with cartoon of representative aortic root aneurysm. B, ERK activation in aortic root samples from MFS patients and donor control and quantification of pERK1/2 relative to vinculin. C, Comparison of ERK activation from aortic root (R) and ascending aorta (A) in individual MFS patients with quantification of pERK activation for individual MFS patients. ***denotes *P* < .01

Marfan syndrome patients with focal aortic root aneurysms (4.67 ± 0.09 cm), normal ascending aortic diameters (2.94 ± 0.14 cm) and an average age 26 ± 3.0 years were selected for comparison to organ donor control patients aged 38 ± 5.5 years (Figure 1A). While multiple groups have noted up‐regulated canonical TGF‐β signalling in MFS human aortic specimens by detecting elevated phosphorylated Smad (pSmad),^25^, ^26^ ERK signalling has not been methodically studied. We compared phosphorylated ERK (pERK1/2) in aortic root specimens from MFS patients (n = 5) with normal donor aortic root controls using a validated semi‐quantitative automated protein blotting system^27^ (Figure 1B). Marfan syndrome aortic root aneurysms exhibited profound pERK1/2 up‐regulation vs donor roots (17.4‐fold, *P* = .012). We also compared aneurysmal aortic root tissue to non‐dilated ascending aortic tissue (‘MFS Asc’ in figures and legends) in MFS patients (Figure 1C). Interestingly, ERK phosphorylation was similarly elevated in both aortic regions (n = 6) from MFS patients (1.06‐fold, *P* = .80), indicating that ERK activation does not correlate with aneurysm formation.

### 3.2 Analysis of SMC phenotype demonstrates enhanced expression of both contractile and synthetic genes in MFS aortic aneurysms, featuring a ‘mixed’ phenotype

**Figure 2:**
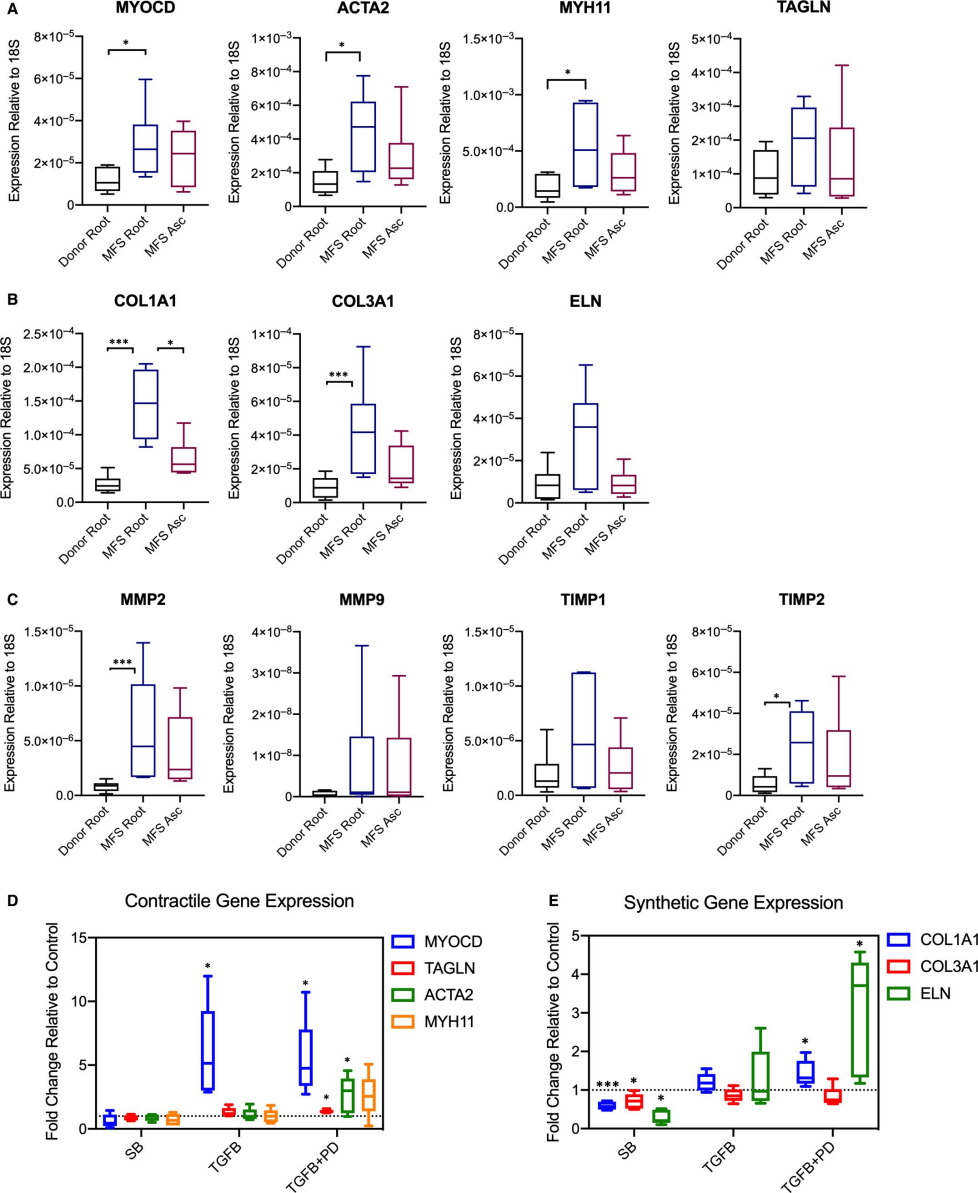
MFS aortic tissues demonstrate enhanced contractile and synthetic gene expression. A, RT‐PCR data for myocardin (MYOCD), a transcriptional regulator of contractile gene expression and downstream genes ACTA2, MYH11 and TAGLN, B, Collagen‐1, Collagen‐3, and elastin expression and C, MMP and TIMP isoforms in MFS aortic tissue specimens compared with donor controls. All data expressed as ratio to expression of 18S, a housekeeping ribosomal RNA using the deltaCT method. n = 6 aortic specimens per group. D, RT‐PCR data for contractile and E, collagen/elastin genes (n = 5 cell lines) displayed as fold change of denoted treatment relative to control media (standard SMC media). *P < .05, ***P < .01

Vascular SMCs play a key role in maintaining the integrity and regulation of the aortic wall. Smooth muscle cells maintain plasticity to evolve from a quiescent, contractile phenotype towards a synthetic phenotype, characterized by proliferation, migration and extracellular matrix (ECM) remodelling.^28^ TGF‐β regulates SMC differentiation during embryonic development and participates in postnatal phenotypic switching.^29^, ^30^ Crosas‐Molist et al^19^ previously characterized phenotypic changes in MFS patients, revealing increased levels of contractile proteins and collagen I via histologic and protein‐based methods. RT‐PCR of our MFS aortic root aneurysm tissue (tunica media) demonstrated a 2.4‐fold increased expression of myocardin (MYOCD) relative to non‐dilated donor control aortic roots (n = 6, *P* = .015, Figure 2A). Because MYOCD is a major transcriptional activator that induces SMC differentiation towards a contractile phenotype,^31^ we next assessed MYOCD‐inducible contractile proteins and found a significant increase in the expression of the SMC‐specific contractile genes myosin heavy chain 11 (MYH11) (3.05‐fold, *P* = .025) and smooth muscle alpha (α)‐2 actin (ACTA2) (2.93‐fold, *P* < .01), but not smooth muscle protein 22‐alpha (SM22‐alpha/TAGLN) (1.88‐fold, *P* = .22). Importantly, we did not find any statistically significant differences in the tested contractile genes with paired comparisons of MFS aneurysmal aortic root vs the same patient's non‐dilated ascending aorta.

Synthetic genes related to ECM remodelling were also tested. Collagen isoforms 1 and 3 (COL1A1 and COL3A1) were both significantly increased in MFS aortic root compared to non‐dilated donor control aortic root (5.6‐ and 5.3‐fold, *P* < .01), while elastin (ELN) expression was not significantly different (3.6‐fold, *P* = .13, Figure 2B). Of these genes, only COL1A1 demonstrated a significant difference in expression level between the aneurysmal MFS aortic root and the same patient's non‐dilated ascending aorta (2.25‐fold higher in aortic root, *P* = .03). MMP2 expression was 5.7‐fold higher in MFS aortic root compared to donor (*P* < .01, Figure 2C); there was no significant difference in MMP9 or TIMP1 expression. TIMP2 was significantly enhanced in MFS aortic root (4.2‐fold, *P* = .04). There were no statistically significant differences between MFS aortic root and non‐dilated ascending aorta for these genes.

Taken together, these results demonstrate that in MFS aortic root aneurysm specimens, both contractile and synthetic (ECM remodelling) gene pathways are activated suggesting a ‘mixed’ tissue phenotype.

### 3.3 Smad and ERK signalling compete for regulation of MFS SMC contractile and ECM genes, in vitro

To further delineate the roles of Smad and ERK signalling in directing SMC gene expression, primary aortic root SMC lines derived from MFS patients (n = 5) were treated with either (a) TGF‐β inhibitor (SB431542, 10 μmol/L), (b) TGF‐β (10 ng/mL) or (c) TGF‐β with ERK inhibitor (PD98059, 20 μmol/L) and compared to control media. TGF‐β inhibition did not significantly affect contractile gene expression. While TGF‐β substantially induced MYOCD overexpression (5.1‐fold increased vs control, *P* < .01 Figure 2C), SMC contractile genes TAGLN, ACTA2 and MYH11 remained at baseline with only TGF‐β. Co‐treatment with TGF‐β and ERK inhibitor, however, led to statistically significant increases in ACTA2 and TAGLN expression (2.3‐ and 1.4‐fold, respectively, *P* < .05). The synthetic genes COL1A1, COL3A1 and ELN were (Figure 2D) significantly reduced following TGF‐β inhibition (0.58‐, 0.68‐ and 0.25‐fold, respectively, *P* < .05). As with contractile genes, TGF‐β treatment did not significantly affect expression, but combined with ERK inhibition significantly increased COL1A1 and ELN expression (1.4‐ and 2.6‐fold, *P* < .05). These results suggest that canonical TGF‐β‐Smad and non‐canonical ERK signalling may exert opposing effects on the tested SMC contractile and synthetic genes. Interestingly, canonical TGF‐β‐Smad signalling appears to promote both contractile and synthetic gene expression, thereby directing SMCs towards the ‘mixed’ phenotype changes observed in MFS aneurysm specimens. ERK acts as an apparent inhibitor of these processes as demonstrated by increased expression in most of the tested genes with ERK inhibition.

### 3.4 Single‐cell RNA‐seq reveals enhanced multiple SMC clusters and increased fraction of synthetic SMCs in MFS

Our tissue and in vitro studies revealed enhancement of gene expression pathways related to both SMC synthetic and contractile function. Because SMCs behave in a spectrum of activity between these pathways, 32 the observed phenotype noted in bulk tissue RT‐PCR could be the result of either a (a) mixed phenotype within single SMCs or (b) discordant behaviour of separate subpopulations. To test this conundrum, we subjected primary aortic root SMC lines from a 41‐year‐old organ donor without history of cardiovascular disease and a 25‐year‐old male MFS patient undergoing surgery for a 4.4 cm aortic root aneurysm to high depth single‐cell RNA sequencing (scRNA‐seq). These individuals were selected as representatives of the MFS and control cohorts for the study (Figure 1A). The data sets were analysed using the Seurat^33^ platform.

**Figure 3:**
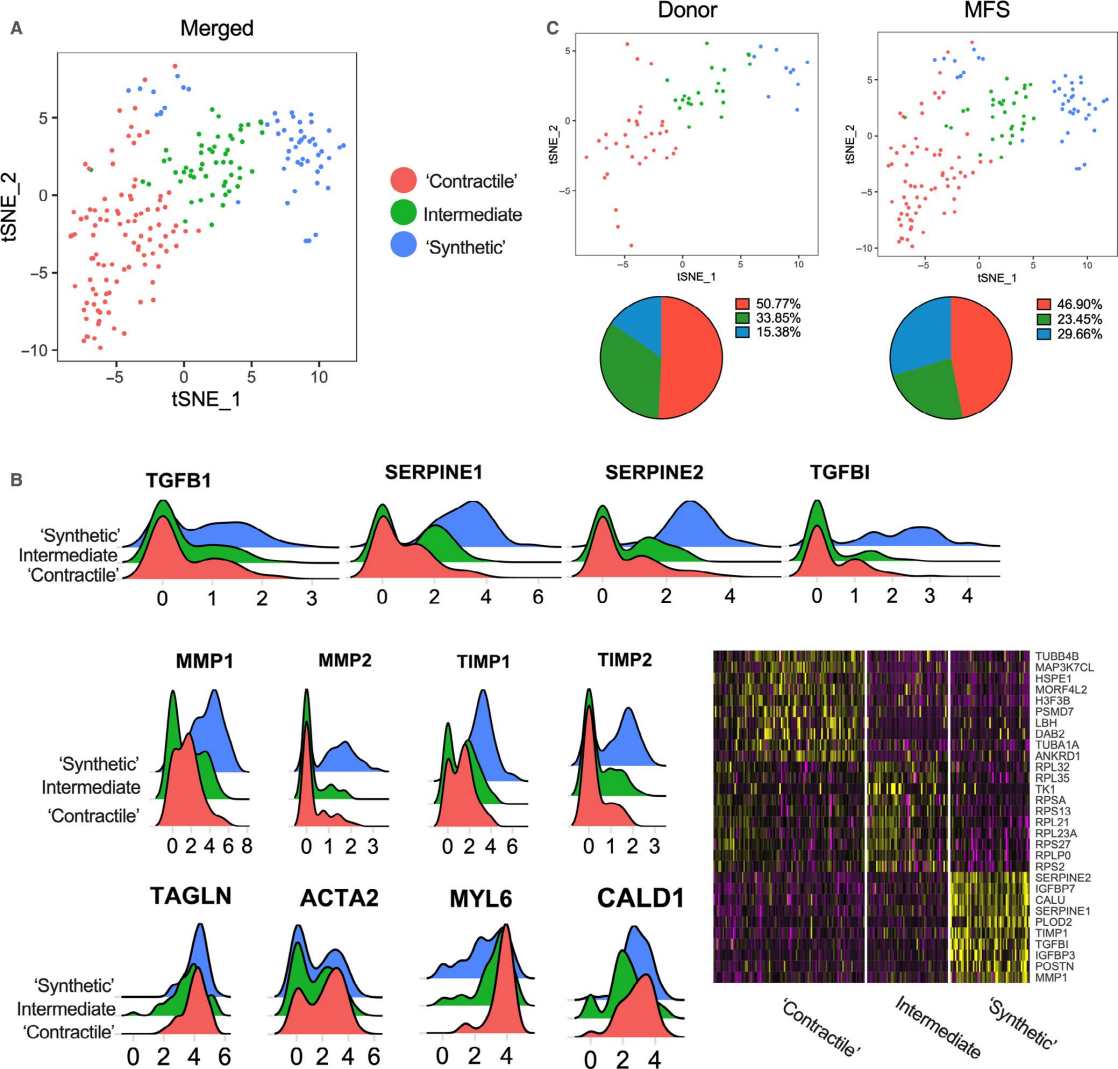
Single‐cell RNA sequencing reveals distinct SMC subpopulations. A, tSNE plot of MFS and donor SMCs demonstrating three distinct clusters. B, Ridge plots and heat map depicting variable expression of TGF‐β‐induced, ECM turnover and contractile genes in the SMC clusters. C, Unmerged ridge plots and cell counts depicting enhanced fraction of synthetic SMC in the MFS sample

Principal component analysis and unsupervised graph clustering of the combined MFS and control data sets revealed several distinct clusters of SMCs (Figure 3A). One cluster was distinguished by increased expression of the canonical TGF‐β responsive genes, SERPINE1 and TGFBI, ECM remodelling genes from both MMP and TIMP families, and reduced expression of SMC contractile markers, consistent with a synthetic SMC phenotype (Figure 3B). The other two clusters demonstrated graded increases in contractile gene expression and decreasing ECM remodelling genes, consistent with intermediate and contractile phenotypes. These data suggest a continuum of expression patterns in SMCs. Separating out the MFS and control patient samples for individual analysis revealed increased fraction of these ‘synthetic’ SMCs in the MFS patient sample compared to donor control (29.7% vs 15.3%, Figure 3C). Therefore, in contrast to traditional quantitative gene expression analysis, single‐cell RNA‐seq reveals distinct and not ‘mixed’ SMC phenotypic clusters in MFS with an increased fraction of synthetic vs contractile phenotypes.

### 3.5 ERK affects critical gene pathways specific to MFS aortic root SMCs including Notch signalling

**Figure 4:**
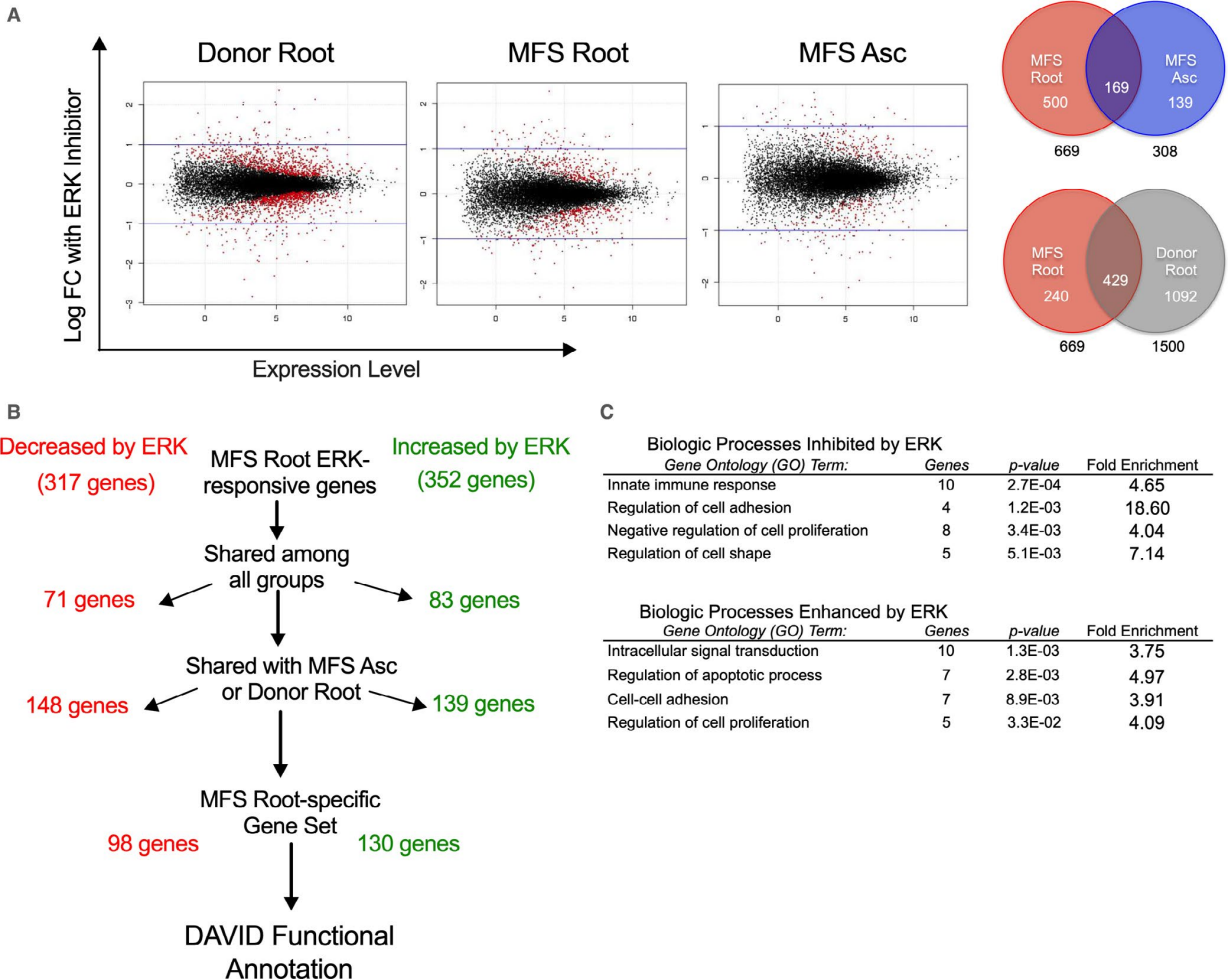
RNA‐sequencing transcriptome‐wide assessment with ERK inhibition. A, Volcano plots for whole‐genome sequencing data depicting expression fold change with ERK inhibition relative to control and quantification of genes with significantly altered expression with ERK blockade at cut‐off *P* < .05. n = 5. B, Workflow for identification of gene set with ERK responsive genes specificity to MFS aortic root. C, DAVID analysis of MFS Root‐specific gene set (228 genes) stratified by biologic processes modulated with ERK blockade. RNA‐sequencing data acquired from n = 5 donor and MFS aortic specimens

To better understand the role ERK signalling plays during aortic root aneurysm development, we performed transcriptome‐wide RNA sequencing on donor control aortic root and MFS (a) aortic root and (b) ascending aorta‐derived SMCs treated with either ERK inhibitor or control media (n = 5 patients in each group). Gene expression changes following ERK inhibitor treatment were assessed for each sample group (Figure 4A). We identified a subset of 228 genes affected by ERK inhibition in MFS aortic root, specifically (Figure 4B). Functional annotation using DAVID software was utilized to identify biologic processes disproportionately represented by these genes. This analysis suggested that ERK may (a) reduce inflammation, (b) modulate apoptosis and (c) enhance proliferation in aneurysmal MFS root SMCs (Figure 4C, complete list of pathways in Table S1). These data imply complex contributions of ERK to SMC turnover in the aortic wall and a complex response related to cell‐cell adhesion, with genes related to these pathways both significantly up‐ and down‐regulated by ERK inhibition.

**Figure 5:**
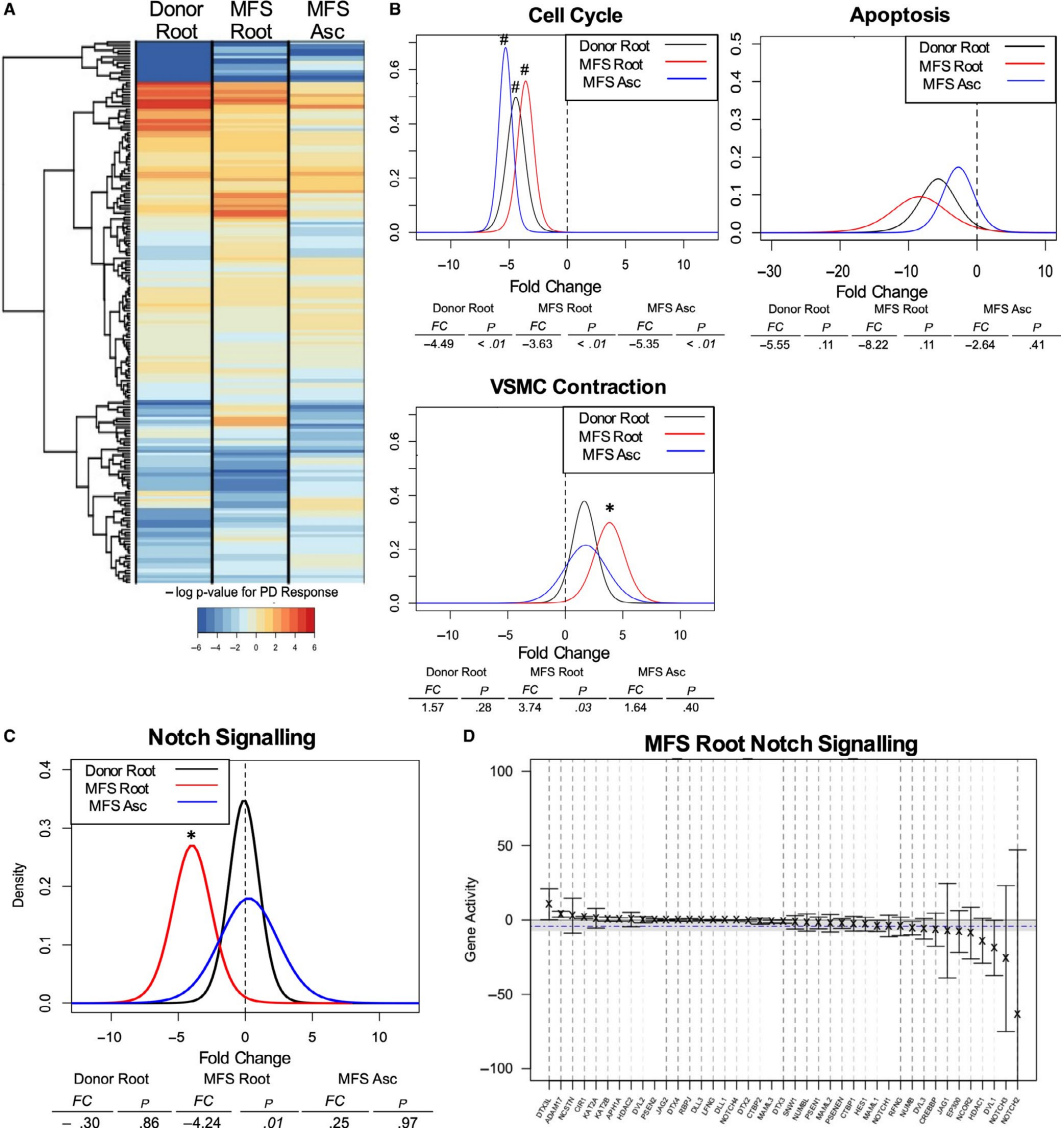
QuSAGE analysis of aortic RNA‐sequencing data identifies Notch signalling as pathway of interest. A, Hierarchical map of 186 predefined KEGG gene sets clustered by ‐log *P* value following QuSAGE analysis; sign denoted positive or negative based on direction of gene set activity change. B, Individual probability density function (PDF) charts developed for pathways of interest relevant to VSMC phenotype. C, PDF plot for Notch signalling pathway and D, summary plot of individual gene activity within Notch signalling pathway following ERK inhibition. *P < .05, #*P* < .01

Having evaluated individual gene regulation in the MFS aortic root only, we next used QuSAGE^34^ (Quantitative Set Analysis for Gene Expression) to systematically assess for changes in core biologic processes in response to ERK. Predefined KEGG gene set responses were assessed for statistical significance and hierarchically clustered (Figure 5A, complete data set in Table S2), revealing differential responses to ERK blockade between groups. To study ERK signalling in processes related to SMC function and aortic aneurysm biology, we specifically evaluated cell cycle, apoptosis and SMC contraction (Figure 5B). All sample groups demonstrated statistically significant reduction in the cell cycle gene set (3.63‐5.35‐fold reduction, *P* < .01), indicating that ERK is a pro‐proliferative signal. All groups trended towards decreased apoptotic gene set expression with ERK inhibitor, although this did not reach statistical significance (2.6‐ to 8.2‐fold reduction, *P* = .11‐.41). Interestingly, ERK inhibitor treatment resulted in a statistically significant increase in SMC contractile genes only in MFS aortic root‐derived SMCs (3.74‐fold *P* = .03). This gene set trended towards enhancement in donor aortic root and MFS ascending aortic SMC samples but was not statistically significant. Accordingly, ERK signalling drives expression of proliferative genes in SMC, although this response was not specific to diseased specimens. Simultaneously, ERK also acts as a silencer of SMC contraction genes. These data support our prior in vitro data in which ERK acts as a ‘brake’ on Smad‐induced contractile gene expression in MFS aortic root vascular SMC and highlight the important role of ERK as a proliferative signal in SMC in general.

QuSAGE analysis was also used to probe for novel pathways of interest by identifying gene sets differentially modulated in the MFS aortic root, the site of aneurysm development. The Notch signalling pathway was reduced 4.24‐fold (*P* = .014) in MFS aortic root SMC with ERK inhibitor, while the donor root and MFS ascending SMCs were unaffected (Figure 5C). Single‐gene‐level analysis of this response suggested changes in expression of Notch receptors and ligands as a driver for this significant response (Figure 5D).

### 3.6 ERK activation increases Notch3 expression in MFS aortic aneurysms

**Figure 6:**
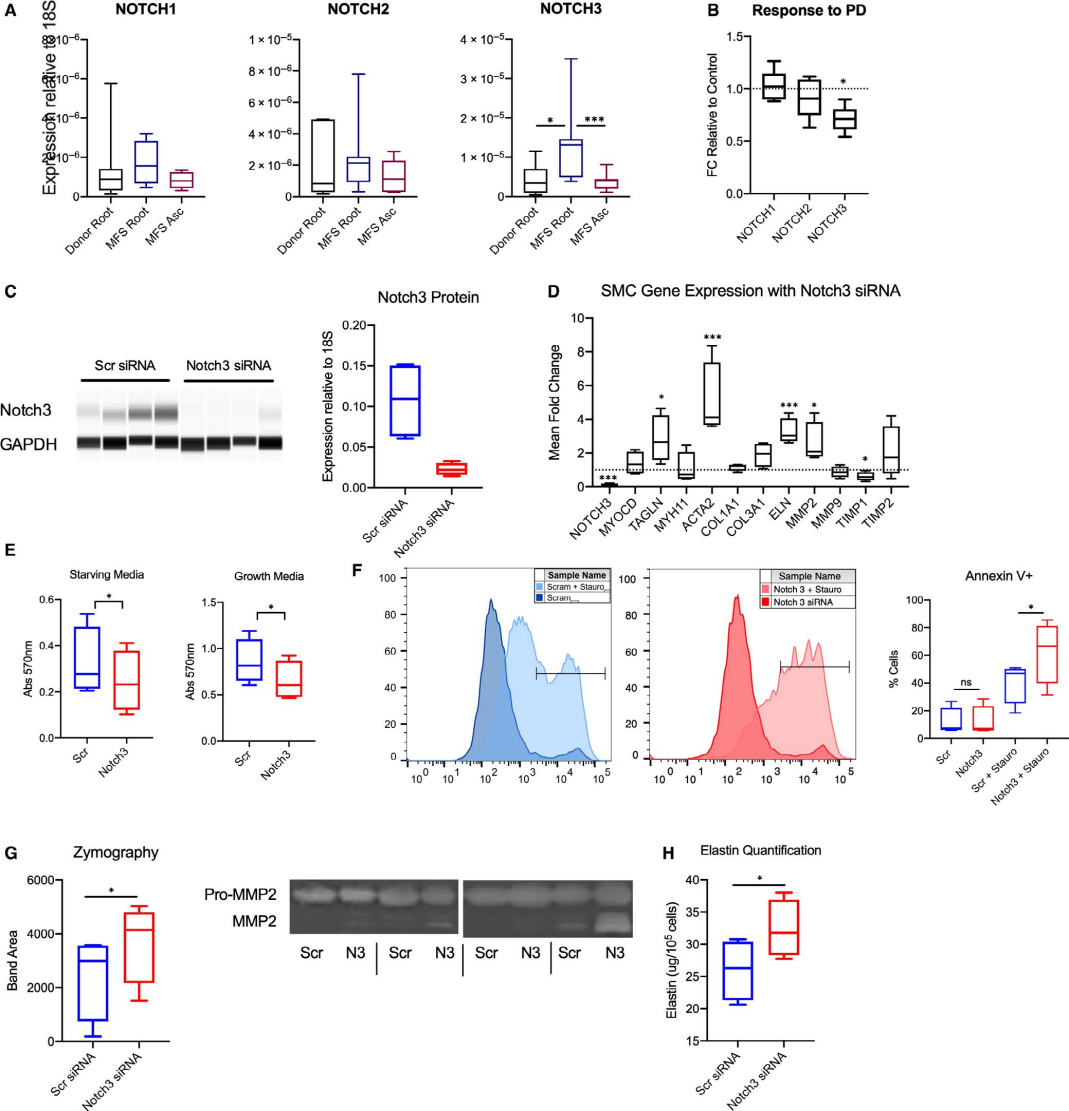
Notch3 expression is enhanced in MFS aortic root; selective Notch3 knockout alters critical SMC phenotype pathways. A, RT‐PCR from n = 8 MFS and donor control aortas for Notch 1‐3 ligand expression normalized to 18S ribosomal RNA. B, Quantitative in vitro assessment of Notch ligand expression following ERK inhibition with PD98059. *P < .05 ***P < .01. C, Immunoblotting and quantification for Notch3 compared to GAPDH as loading control. D, RT‐PCR for Notch3 expression and critical SMC genes depicted as fold change relative to scrambled siRNA. E, MTT raw data in starving and growth media conditions. F, Representative flow cytometry histogram for Annexin V staining and quantitative analysis in Notch3 and scrambled siRNA groups with and without staurosporine as stimulus for apoptosis. G, Gelatin zymography quantification and representative zymogram. H, Total elastin/tropoelastin content measured with colorimetric assay. All assays completed with n = 4 MFS aortic root SMC lines subjected to liposome‐based transfection of scrambled or Notch3 siRNA. *P < .05 ***P < .01 for paired *t* test compared to scrambled siRNA control

Notch signalling is a critical component of aortic valve development and VSMC development and differentiation, while perturbations in Notch signalling have been described in aortopathy related to bicuspid aortic valve.^22^ Given the novel finding that ERK enhances the Notch signalling pathway exclusively in aneurysmal MFS aortic root‐derived SMCs in vitro, we subsequently examined the expression of Notch receptors in MFS and non‐dilated donor aortic surgical specimens (n = 8 patients). We identified significant enhancement of Notch3 receptor expression in MFS aortic root specimens relative to both the non‐dilated MFS ascending aorta and donor aortic root (3.05‐fold and 3.48‐fold, respectively, *P* < .05). Notch1 and 2 expression were not significantly different between MFS and donor aortic root specimens (Figure 6A).

We next performed RT‐PCR on primary aortic SMC cell lines treated with PD98059 or vehicle control for 24 hours (n = 5). Marfan syndrome aortic root SMCs demonstrated a significant reduction in Notch3 expression with ERK blockade, whereas Notch1 and 2 did not significantly change (Figure 6B). Therefore, these in vitro studies suggest that focal Notch3 overexpression in the aneurysmal MFS aortic root may be ERK‐dependent.

### 3.7 Notch3 enhances aortic root SMC proliferation, reduces apoptosis and modulates ECM turnover, in vitro

To better understand the role Notch3 may play during aneurysm formation, MFS aortic root SMC lines (n = 4 patients) were treated with a Notch3‐specific siRNA or scrambled control (Scr), then tested for SMC‐specific gene expression and functional phenotypic changes. Effective knockdown at both the protein (4.7‐fold, *P* = .03) and RNA level (9.7‐fold reduction, *P* = .02) were verified by protein immunoblotting and RT‐PCR, respectively (Figure 6C).

Changes in important SMC phenotypic genes were assessed in response to Notch3 silencing (Figure 6D). Notch3 siRNA increased TAGLN and ACTA2 gene expression (2.56‐fold, *P* = .04 and 4.74‐fold, *P* < .01, respectively), while MYOCD and MYH11 were unaffected. When testing components of the ECM, ELN expression was up‐regulated (3.21‐fold, *P* < .01), yet COL1A1 and COL3A1 expression levels remained unchanged. Intriguingly, MMP2 expression was 2.39‐fold increased (*P* = .024) with Notch3 knockdown while TIMP1 expression was reduced (0.50‐fold expression, *P* = .019). MMP9 and TIMP2 expression were not significantly altered.

Cell‐based assays were used to further characterize the effects of Notch3 on aortic root SMCs. Cell proliferation was assessed via MTT assay. Notch3 siRNA inhibition resulted in 25% MTT reduction in both starved and normal media (*P* < .05 for both conditions), suggesting Notch3 naturally increases cell proliferation (Figure 6E). Apoptosis was studied using a flow cytometry based assay for cell surface Annexin V (Figure 6F). In control conditions, no difference was detected in apoptosis (Annexin V staining) between Scr and Notch3 siRNA groups (11.9 ± 5.0% vs 12.2 ± 5.5%, *P* = .63). However, when Staurosporine treatment was used to induce apoptosis, Notch3 knockdown resulted in substantially higher Annexin V staining (63.3 ± 11.3% vs 40.8 ± 7.5%, *P* = .017). Finally, ECM turnover in response to Notch3 silencing was tested. Gelatin zymography from conditioned cell media demonstrated significantly more MMP2 activity (3.12 ± 1.7‐fold, *P* = .014) with Notch3 knockdown (Figure 6G). Elastin and tropoelastin synthesis were quantified with a cell lysate‐based assay and demonstrated modest but significant increase in ELN production with Notch3 silencing (24% increase, *P* = .013, Figure 6H). These in vitro studies reveal that Notch3 likely reduces aortic root SMC contractile protein expression while enhancing cell proliferation, resisting apoptotic stimuli and decreasing MMP activity in MFS aortic root SMCs. While these changes suggest a largely protective role for Notch3 with regards to aneurysm biology, Notch3 may act as an inhibitor of ELN expression in vitro*.*

## 4 DISCUSSION

The pathophysiology of aneurysm development in MFS is a complex, multi‐factorial process. TGF‐β is central to this process; however, its effects appear highly dependent on developmental stage. Further complicating matters, TGF‐β signalling proceeds through multiple downstream pathways, including ERK. While ERK signalling has been implicated as a driver of aneurysm pathogenesis in the Fbn1^C1039G/+^ MFS model,^15^, ^35^ ERK activation was significantly enhanced with losartan and anti‐TGF‐β therapy and coincided with aneurysm prevention in the more aggressive Fbn1^mgR/mgR^ mouse model.^36^ Furthermore, ERK inhibition exacerbated SMC pathology in an induced pluripotent stem cell‐derived model of MFS by increasing apoptosis and reducing proliferation.^37^ Importantly, whether ERK signalling drives aneurysm formation in the human disease has not been firmly established. Utilizing both human aortic specimens and primary cell culture lines, we systematically assessed ERK signalling in the context of SMC phenotype in MFS aortic root aneurysms. The major findings of this study include the following: (a) ERK is uniformly activated in MFS aortic root and non‐dilated ascending aorta; (b) MFS aortic SMCs demonstrate increased heterogeneity characterized by both increased contractile and synthetic gene pathways in parallel; and (c) ERK does not drive these disturbances and may have beneficial effects including proliferation, dampening of TGF‐β‐induced changes and crosstalk with Notch3, which appears to play a protective role.

Our data demonstrate that ERK is robustly activated in both the MFS aneurysmal aortic root, as well as the normal calibre ascending aorta. Enhanced Smad2 activation in both aneurysmal and non‐dilated aorta has been previously reported,^19^ although Smad2 activation was more pronounced in the dilated aortic root using histopathologic techniques. Lineage studies have shown that vascular SMCs in the different aortic anatomic segments have distinct embryologic origins.^38^ Recent reviews have suggested that the diversity of SMC origin may explain site‐specific location of various diseases, including aneurysm formation.^39^ Under this assumption, if enhanced ERK signalling contributes to MFS aneurysm formation, SMCs that originate from the aortic root (second heart field) must respond differently following ERK activation compared to SMC from the ascending aorta (neural crest). Our surgical specimens are intrinsically limited by analysis late in the disease process; therefore, the contribution of ERK to aneurysm development at earlier time‐points remains unclear.

Phenotypic ‘switching’ of aortic constituent cells contributes to aneurysm formation in abdominal,^40^, ^41^ descending thoracic^42^ and aortic root aneurysms.^43^ This study confirms the *simultaneous* enhancement of both contractile and synthetic gene sets in MFS proposed by Crosas‐Molist et al^19^ who similarly observed an overexpression of both contractile markers and collagen I in human MFS specimens. Our investigation of SMC phenotype in this context implicates canonical TGF‐β‐Smad signalling, rather than the non‐canonical ERK pathway, as the driver of the detected changes. RNA sequencing at the single‐cell level revealed distinct SMC gene expression signatures, suggesting that the ‘mixed’ phenotype observed in MFS aortic tissue is a conglomeration of multiple distinct SMC phenotypes in the aortic wall, with a larger proportion of SMCs reprogrammed into the synthetic phenotype. We hypothesize that complex dysfunctional interactions among the noted distinct SMC populations participate in MFS aneurysm formation. The mechanisms underlying the heterogeneous fate of SMCs in response to prolonged TGF‐β stimulation present a provocative area for further research. This finding is in contrast to the pure synthetic SMC phenotype noted in the chemically induced calcium chloride descending aneurysm model^33^. Distinctively, we discover MYOCD, MYH11 and ACTA2 contractile genes similarly increased in the nearby non‐aneurysmal aorta. Of the genes screened, only COL1A1 gene expression was specifically enhanced in the aneurysmal aortic root. Whether collagen deposition accelerates aneurysm formation or represents a beneficial response to reinforce the aortic wall continues to be controversial. While collagen has been proposed to increase aortic stiffness and predisposition to aneurysm and dissection,^44^ Busnadiego et al^45^ demonstrated that reduction of mature collagen deposition via lysyl oxidase enzyme blockade worsens aneurysm severity in a murine model. In patients, Baumgartner et al^46^ report that high aortic wall distensibility was a favourable prognostic indicator, whereas reduced elasticity was predictive of aortic wall abnormalities in MFS patients.

**Figure 7:**
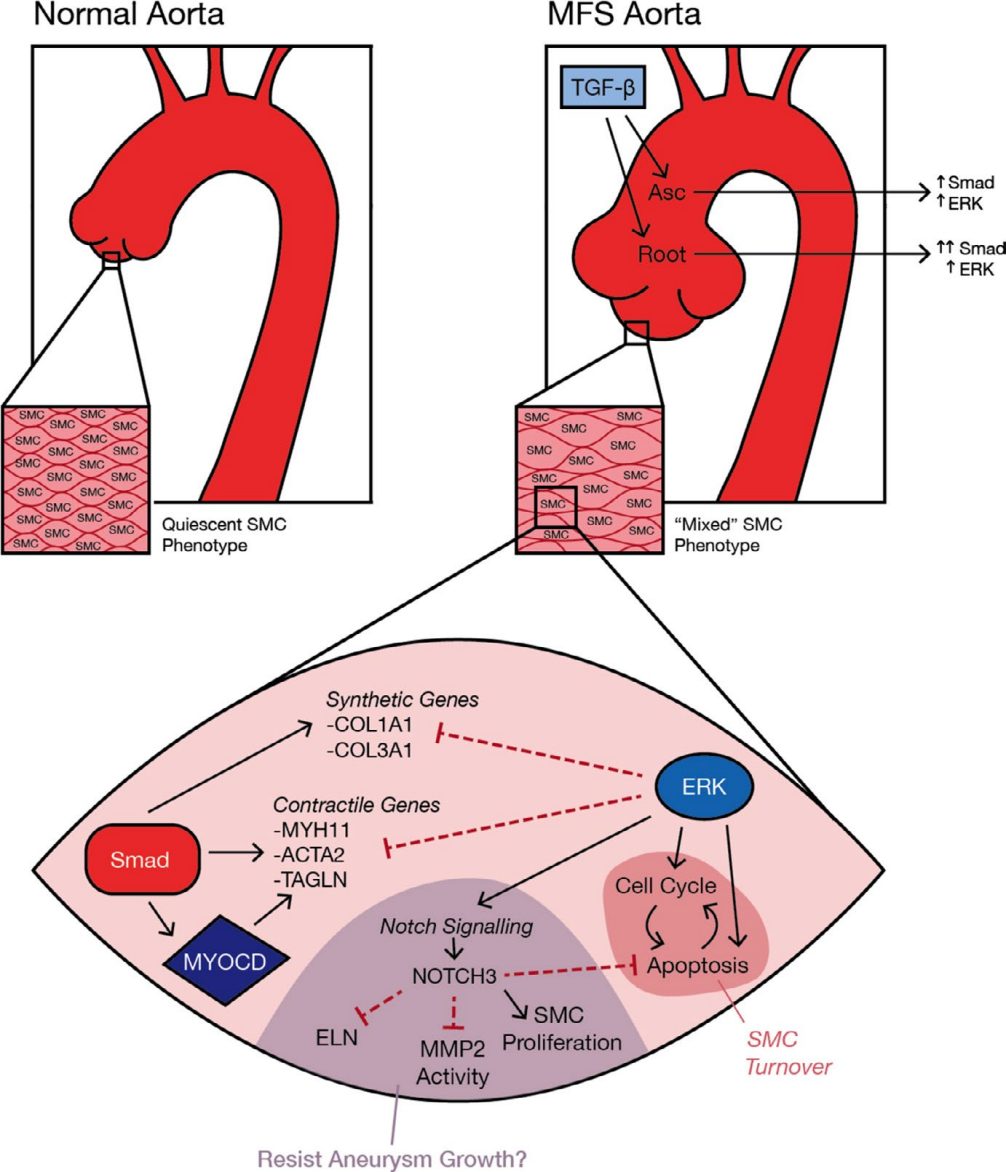
Proposed role of TGF‐β‐dependent ERK/Smad signalling in MFS aortic root aneurysms. SMCs in the aortic root and ascending aorta receive enhanced TGF‐β signalling. While Smad signalling is more enhanced in the aneurysmal aortic root compared to the non‐dilated ascending aorta, ERK activation is uniformly increased in both regions. Normal quiescent contractile SMCs may adopt increased contractile/synthetic gene expression leading to heterogeneity and ‘mixed’ phenotype in the aortic wall, likely driven by Smad signalling. ERK dampens Smad‐dependent changes and seems to play a role in SMC turnover by enhancing cell cycle‐related genes and apoptosis. NOTCH3 is focally enhanced by ERK in aneurysmal MFS aortic root SMCs and may represent a protective response against further aneurysm growth

Hypothesizing that Smad is the dominant signalling pathway influencing SMC phenotype late during MFS aneurysm formation, we sought to characterize the downstream effects of ERK activation. RNA sequencing following ERK inhibition was utilized as a tool to (a) establish ERK‐dependent aortic root processes in general (normal vascular SMC) and (b) determine ERK‐specific responses in the MFS aortic root (pathologic vascular SMC). Phenotypically normal donor SMC groups clearly demonstrated that ERK is a robust activator of proliferation. The pathologic MFS aortic root SMCs revealed an ERK‐dependent reduction in SMC contractile genes. Further analysis illustrated that ERK signalling may affect SMC contractile gene expression, apoptosis, cell adhesion and anti‐inflammatory pathways specifically in the aneurysmal MFS aortic root (Figure 7). A key question remains how ERK influences SMC gene expression over time relative to other TGF‐β‐dependent pathways.

The Notch signalling pathway was initially discovered to be ERK‐dependent in MFS aortic root‐derived SMCs. Notch3 expression was found to be ERK responsive and subsequently confirmed to be up‐regulated in human MFS aneurysmal aortic root surgical specimens. Of the four Notch receptor subtypes, Notch1‐3 are located on SMCs and critical for development, homeostasis and response to injury.^47^ In the current study, both in vitro and tissue data support the association of enhanced ERK and Notch3 expression. Given the novel finding that Notch3 is up‐regulated specifically in MFS aortic aneurysm specimens, our initial theory was that this pathway might contribute to focal aneurysm development. Surprisingly, Notch3 overexpression appears to confer adaptive responses against aneurysm growth, including inhibition of SMC contractile gene overexpression, promotion of SMC proliferation, reduction of apoptosis and decreased MMP activity. These expression changes are generally consistent with the effects of Notch3 overexpression in pulmonary arterial SMCs^48^, ^49^ and with in vitro models by investigators exploring the effect of Notch signalling on vascular SMCs.^50^, ^51^

The nuance of spatiotemporal TGF‐β signalling leading to aortic root aneurysms in MFS has precluded the development of novel therapies to prevent or slow aneurysm growth. The novel findings of this study include the following: (a) ERK signalling is not synergistic with canonical TGF‐β signalling with regard to SMC phenotype in MFS; (b) SMCs receive a complex combination of TGF‐β‐induced signals ultimately producing a mixed synthetic‐contractile phenotype that likely disrupts aortic homeostasis; and (c) ERK‐dependent Notch3 overexpression may be an intrinsic protective response against aneurysm growth. This novel potential tissue remodelling pathway is a provocative avenue for therapeutic intervention via forced early Notch3 overexpression. Finally, the therapeutic effects of TGF‐β blockade in MFS are clearly time‐dependent and convoluted given that ERK vs Smad signalling may be detrimental at distinct stages of aneurysm formation. Intriguingly, late blockade may be ineffective and perhaps even harmful by reducing the protective ERK‐dependent Notch3 axis.

This study has several limitations. The use of human patient samples and cultured SMC lines intrinsically limits our analysis to the biology of developed aneurysms in MFS. Secondly, because we focus on transcriptomic changes downstream of TGF‐β and ERK, it remains possible that protein quantification would reveal unique findings. Finally, our conclusion that distinct cell populations operate in MFS aortic aneurysms is based on in vitro single‐cell data and tissue characterization. Further characterization of cell lines and primary surgical tissue from additional patients will provide clarity to the roles of these distinct cell populations in aortic aneurysms.

## CONFLICT OF INTEREST

The authors confirm that there are no conflicts of interest.

## AUTHOR CONTRIBUTIONS

AJP and MPF designed the project and wrote the manuscript, AJP, TK, JT, IP, KJ and GB performed experiments and analysed data, AR performed analysis of computational data, PC provided critical assistance acquiring donor tissue, YT, JC and CI assisted with data analysis and critical paper assessment, and GB assisted with pathologic tissue analysis.

## Data Availability

The data that support the findings of this study are available from the corresponding author upon reasonable request.
